# Bioinformatics in microbial biotechnology – a mini review

**DOI:** 10.1186/1475-2859-4-19

**Published:** 2005-06-28

**Authors:** Arvind K Bansal

**Affiliations:** 1 Department of Computer Science, Kent State University, Kent, OH 44242, USA

## Abstract

The revolutionary growth in the computation speed and memory storage capability has fueled a new era in the analysis of biological data. Hundreds of microbial genomes and many eukaryotic genomes including a cleaner draft of human genome have been sequenced raising the expectation of better control of microorganisms. The goals are as lofty as the development of rational drugs and antimicrobial agents, development of new enhanced bacterial strains for bioremediation and pollution control, development of better and easy to administer vaccines, the development of protein biomarkers for various bacterial diseases, and better understanding of host-bacteria interaction to prevent bacterial infections. In the last decade the development of many new bioinformatics techniques and integrated databases has facilitated the realization of these goals. Current research in bioinformatics can be classified into: (i) *genomics *– sequencing and comparative study of genomes to identify gene and genome functionality, (ii) *proteomics *– identification and characterization of protein related properties and reconstruction of metabolic and regulatory pathways, (iii) cell visualization and simulation to study and model cell behavior, and (iv) application to the development of drugs and anti-microbial agents. In this article, we will focus on the techniques and their limitations in genomics and proteomics. Bioinformatics research can be classified under three major approaches: (1) analysis based upon the available experimental wet-lab data, (2) the use of mathematical modeling to derive new information, and (3) an integrated approach that integrates search techniques with mathematical modeling. The major impact of bioinformatics research has been to automate the genome sequencing, automated development of integrated genomics and proteomics databases, automated genome comparisons to identify the genome function, automated derivation of metabolic pathways, gene expression analysis to derive regulatory pathways, the development of statistical techniques, clustering techniques and data mining techniques to derive protein-protein and protein-DNA interactions, and modeling of 3D structure of proteins and 3D docking between proteins and biochemicals for rational drug design, difference analysis between pathogenic and non-pathogenic strains to identify candidate genes for vaccines and anti-microbial agents, and the whole genome comparison to understand the microbial evolution. The development of bioinformatics techniques has enhanced the pace of biological discovery by automated analysis of large number of microbial genomes. We are on the verge of using all this knowledge to understand cellular mechanisms at the systemic level. The developed bioinformatics techniques have potential to facilitate (i) the discovery of causes of diseases, (ii) vaccine and rational drug design, and (iii) improved cost effective agents for bioremediation by pruning out the dead ends. Despite the fast paced global effort, the current analysis is limited by the lack of available gene-functionality from the wet-lab data, the lack of computer algorithms to explore vast amount of data with unknown functionality, limited availability of protein-protein and protein-DNA interactions, and the lack of knowledge of temporal and transient behavior of genes and pathways.

## Background

In the last decade, the revolution in computer technology and memory storage capability has made it possible to model grand challenge problems such as large scale sequencing of genomes and management of large integrated databases over the Internet. This vastly improved computational capability integrated with large-scale miniaturization of biochemical techniques such as PCR, BAC, gel electrophoresis and microarray chips has delivered enormous amount of genomic and proteomic data to the researchers all over the world. This availability of data has led to an explosion of genome and proteome analysis leading to many new discoveries and tools that are not possible in wet-lab experiments.

The availability of genomic and proteomics data and improved bioinformatics and biochemical tools has raised the expectation of the humanity to be able to control the genetics by manipulating the existing microbes. The advantages are enormous such as better diagnosis of the diseases through the use of protein biomarkers, protection against diseases using cost effective vaccines [56,73] and rational drug design, improvement in agricultural quality and quantity, and the development of techniques that help us visualize and understand the detailed microbial machine at the systemic level.

Since the sequencing of the first complete microbial genome of *Haemophilus influenzae *in 1995 [29], hundreds of microbial genomes have been sequenced and archived for public research in GenBank  through the concerted effort of federal health agencies such as NIH and DOE in USA, EMBL and EBI in Europe, DNA databank of Japan, national laboratories, academic universities, multiple drug development companies such as Celera and non-profit organizations such as TIGR, and companies involved in agricultural industry and bioremediation. The sequencing of human genome [68] and other relevant eukaryotic genomes has raised the expectation of understanding host pathogen interaction for the development of better vaccines and rational drugs to control the gene level and pathway level aberrations that are responsible for pathogenesis.

Except for the availability of bioinformatics techniques, the vast amount of data generated by genome sequencing projects would be unmanageable and would not be interpreted due to the lack of expert manpower and due to the prohibitive cost of sustaining such an effort. In the last decade bioinformatics has silently filled in the role of cost effective data analysis. This has quickened the pace of discoveries, the drug and vaccine design [56] and the design of anti-microbial agents [40]. In addition bioinformatics analysis has enhanced our understandings about the genome structure and the microorganism restructuring process.

Bioinformatics analysis will facilitate and quicken the analysis of systemic level behavior of cellular processes, and to understanding the cellular processes in order to treat and control microbial cells as factories. For the last decade, bioinformatics techniques have been developed to identify and analyze various components of cells such as gene and protein function, interactions, and metabolic and regulatory pathways. The next decade will belong to understanding cellular mechanism and cellular manipulation using the integration of bioinformatics, wet lab, and cell simulation techniques. More recently, researchers have started using these techniques for the production of recombinant proteins [48]. It is anticipated that in this decade, the semi-automated study of cellular behavior at systemic level will accelerate this capability.

## Review

In the last decade, Bioinformatics has been used for the microbial biotechnology in many ways: computationally analyzing the wet-lab data, genome sequencing, identification of protein coding segments [6,24,41,64], and genome comparison to identify the gene function [4,5,11,25,35,46,53,70,71], the development of genomic and proteomics databases [8,9,16,21,33,49,62,63], and inference of phenotypes (higher level functions) from genotypes (gene level functions). In order to understand higher level functions four major studies have been undertaken: (i) automated reconstruction and comparison of metabolic pathways [12,14,18,38,49,58,59,65], (ii) study of protein-protein and protein-DNA interactions to understand regulatory pathways [2,7,15,27,28,30,42-44,47,55,60,61,66], (iii) modeling 2D and 3D structure of proteins [10,31,52,57,67], and (iv) modeling the docking of 3D models of proteins with drugs [34]. Understanding 3D structure of proteins has a major impact in understanding protein-protein interactions. Protein-protein and protein-DNA interactions will provide a good understanding of binding sites in signaling pathways; understanding the interactions between proteins and chemical compounds has already facilitated the development of drug design.

Three approaches have been used in bioinformatics: (i) use of computational search and alignment techniques [4,5,53,70] to compare new genome against the set of known genes to annotate the structure and function of genes in a newly sequence genome, (ii) the use of mathematical modeling techniques such as data mining, statistical analysis, neural networks, genetic algorithm, and graph matching techniques to identify common patterns, features and high level functions, and (iii) an integrated approach that integrates search techniques with mathematical modeling.

### Genome sequencing

The major contribution of the bioinformatics in genome sequencing has been in the: (i) development of automated sequencing techniques that integrate the PCR or BAC based amplification, 2D gel electrophoresis and automated reading of nucleotides, (ii) joining the sequences of smaller fragments (contigs) together to form a complete genome sequence, and (iii) the prediction of promoters and protein coding regions of the genome.

PCR (Polymerase Chain Reaction) or BAC (Bacterial Artificial Chromosome) based amplification techniques derive limited size fragments of a genome. The available fragment sequences suffer from nucleotide reading errors, repeats – very small and very similar fragments that fit in two or more parts of a genome, and chimera – two different parts of the genome or artifacts caused by contamination that join end to end giving a artifactual fragment. Generating multiple copies of the fragments, aligning the fragments, and using the majority voting at the same nucleotide positions solve the nucleotide reading error problem. Multiple experimental copies are needed to establish repeats and chimeras. Chimeras and repeats are removed before the final assembly of the genome-fragments. The joining of the fragments is modeled as a mathematical weighted graph where nodes are fragments and the weights of edges are the number of overlapping nucleotides, and the fragments are joined based upon maximum overlap using a greedy algorithm [46,70]. In a greedy algorithm, most nodes having maximum (or minimum) scores are collapsed first. To join contigs, the fragments with larger nucleotide sequence overlap are joined first.

### Automated identification of genes

After the contigs are joined, the next issue is to identify the protein coding regions or ORFs (open reading frames) in the genomes. The identification of ORFs can be done in three ways: (1) using Hidden Markov Model (HMM) based techniques such as GLIMMER [24] and GeneMark [41], (2) by searching the known database of genes such as GenBank  to identify genes, and (3) the use of algorithms based on decision trees that identify start codons [64] and stop codons of the coding regions. HMM based techniques develop multiple probabilistic state machines each capable of identifying an ORF. Each machine predicts the next nucleotide character using a state transition with maximum probability and matches the predicted nucleotide character with the current nucleotide character in the actual sequence. Statistical training using known sample sequences derives the probability of state transition. In the case of microbial genomes, the HMM based software such as GLIMMER has provided 95% – 97% accuracy.

### Identifying gene function: searching and alignment

After identifying the ORFs (open reading frames), the next step is to annotate the genes with proper structure and function. The function of the gene has been identified using popular sequence search and pair-wise gene alignment techniques. The four most popular algorithms used for functional annotation of the genes are BLAST [4] and its variations [5], dynamic programming technique Smith-Waterman alignment [70] and its variations, indexing based scheme FASTA [53] and its variations, and BLOCKS [35] that uses multiple sequence alignment of conserved domains to identify motifs – characterizing patterns of proteins.

BLAST search is based upon expanding multiple probable seed points (longer than four nucleotides) that match (with the help of scoring matrices such as BLOSUM or PAM [46,70]) to identify the largest matching nonrandom segment. Scoring matrices have positive match-value for the amino acids that have common biochemical or biophysical properties and negative match-values if the amino acids do not share biophysical or biochemical properties. Substitution matrices such as BLOSUM (BLOcks SUbstitution Matrix) have been derived by statistically comparing the frequency patterns of the amino acids occurring in conserved domains of protein families. Nucleotide sequences use a nucleotide matrix for scoring that penalizes non-matching positions. BLAST algorithm has near linear time complexity, and the current implementations are fast. However, in order to enhance computational efficiency, BLAST algorithm uses most probable combinations of nucleotide seeds to index the sequences in the database sacrificing some accuracy.

BLAST algorithm has gone through many improvements in heuristics to improve the execution speed, accuracy, and the dependence on predefined scoring matrices. Two major improvements are: (i) the use of two or more hits within a matching region before extending the high scoring segment, and the use of multiple iteration of matching to derive a position specific scoring matrix to be used in place of predefined biochemical matrix. PSI-BLAST (Position Specific Iterative BLAST) [5] is a popular implementation of BLAST that uses both these improvements. The use of two hits improves the execution efficiency in the segment extension, and the use of position specific matrix improves the search for weakly homologous sequences in evolutionary distant species. Position specific matrix is built by deriving multiple sequence alignment of the best matching segments and analyzing the frequency of the amino acid substitutions in the matching segments.

Dynamic algorithms such as Smith-Waterman [70] and other indexing schemes [53] are more accurate for pair-wise gene alignment. The alignment of gene-pairs using dynamic algorithms is based upon incremental matching by maximizing the sum of the score of the best alignment of the preceding subsequences and the score of matching the current amino-acid characters (or nucleotide characters). The mismatches in amino-acid sequences are penalized using scoring matrices such as BLOSUM or PAM [46,70]; the nucleotide sequences use a nucleotide matrix for scoring that penalizes non-matching positions. A gap is inserted to show the insertion and deletion of nucleotides (or amino-acids). Gaps are not part of a substitution matrix, and are provided as parameters by users. The presence of a gap also results into score penalty. There are two major types of protein (or gene) alignments using dynamic programming: global and local. In global alignment, the amino-acid (or nucleotide) characters are placed to maximize the overall score. In contrast local alignment finds the segment with the maximum score, and the segments with negative scores are ignored. For comparing amino acid sequences from evolutionary distant organisms, local alignment is preferred to take care of large-scale amino-acid variations. Global alignment fares well when small amount of random mutation is involved. Due to the pair-wise comparison of all characters in an amino acid sequence to identify best matching subsequence, all dynamic programming techniques have quadratic time complexity making them less suitable for large-scale pair-wise genome comparisons unless preprocessed by BLAST to remove dissimilar genes [11].

Multiple sequence alignment techniques [22] compare multiple homologous genes (genes that have similar sequences) to derive conserved segments and to derive evolutionary tree. The technique uses the integration of pair-wise alignment between two homologs and the notion of distance between two nucleotide sequences or between two amino-acid sequences. The notion of distance can be derived either as an edit distance – number of mismatches derived after pair-wise alignment of two sequences, or as the evolutionary distance between two microorganisms given by an evolutionary tree. The technique is based upon progressive pair-wise comparison to make intermediate alignments between nearest neighbors – homologs having shortest distance, and has been implemented as a greedy algorithm. ClustalX [22] is a popular multiple sequence alignment technique that has been used to identify conserved portions in a gene, and to develop new evolutionary trees [36].

A major source of problem in the above sequence comparison techniques is the assignment of user defined equal weight to indels (gaps) that undermines the importance of a specific amino-acid(s) or a group of amino-acid characters would have. Another minor problem is the presence of repeat characters in the sequences as the repeat characters only show the functional or structural separation of the component units within a gene, and can not be mixed with other amino-acid characters.

Multiple sequence comparison techniques such as BLOCK [35] have been used to identify the conserved subsequences in very similar gene sequences, and are good to derive motifs. Motifs – a set of unique subsequences characterizing a protein – have been found very useful to identify genes with the same functionality. Motifs are derived by identifying the conserved subsequences of the functionally equivalent genes from multiple organisms after aligning the sequences.

Protein domain is the basic unit of protein function and is associated with a unique pattern (possibly one) of folding (alpha helix, beta sheet or their variations) at the structure level. The researchers have used multiple sequence alignment and HMM to identify the regions that are individually homologous to each other in multiple homologous genes. These regions are probable domains. Currently there are many domain related databases such as PRODOM [21], Pfam [16] and SMART [39] (also see ). Pfam [16,63] (and ) is a database of multiple alignments of protein domains or conserved protein regions. The alignments represent some evolutionary conserved structure that has implications for the protein's function. Profile hidden Markov models (profile HMMs) built from the Pfam alignments are useful for automatically recognizing that a new protein belongs to an existing protein family, even under weak homology. Currently Pfam is derived automatically by cluster analysis of PRODOM database.

The sequence search based techniques assume that best sequence is sufficient to annotate the function. This assumption is generally true. However, in many cases best sequence match fails to identify the function due to: (1) function being localized to a specific area in the protein such as hydrophobic region, (2) the function being dependent on the presence of specific pattern of amino acids, or (3) function being dependent to a specific 3D conformational state in a multi domain protein. Sometimes mutation of few nucleotides alters the corresponding amino acids resulting into a different 3D conformation of a protein. Another limitation of best match techniques is that they cannot identify all possible functions of a multi-domain protein. A protein may have multiple domains, and may be multifunctional. The problem is more complex as there is no direct correlation between the number of domains in a protein and the number of functionality [32,37].

### 3D structure modeling and docking

A protein may live under one or more low free-energy conformational states depending upon its interaction with other proteins. Under a stable conformational state certain regions of the protein are exposed for protein-protein or protein-DNA interactions. Since function is also dependent upon exposed active sites, protein function can be predicted by matching the 3D structure of an unknown protein with the 3D structure of a known protein [10,71]. However, 3D structures from X-ray crystallography and NMR spectroscopy are limited. Thus there is a need for alternate mechanism to match genes. Generally there is close correspondence between gene sequence and 3D structure. In such cases sequence matching is sufficient for function annotation. However, many times multiple sequences map to the same 3D structure; the lack of matching of amino acid sequences does not exclude same 3D structure. In such cases matching 2D structure [57,66] – patterns of alpha helix and beta sheets – and matching 3D structures is needed to verify the function of the newly sequenced protein [71].

There are two major approaches to model 3D structure of a protein: (i) sequence homology based prediction and (ii) *ab initio *(or *de novo*) method. The sequence homology approach uses sequence alignment to identify the best matching 3D structure for different components: conserved portion, loop portion and side chains from the database, and threads them to predict the overall 3D structure. The *ab initio *method is based upon energy minimization principle, and predicts the structure from the sequence alone [10]. Recent advances in ab initio methods integrate the biochemical and biophysical properties such as folding of beta sheets and the information of hydrophobic regions to achieve better accuracy.

Docking is a term used to identify best matches between 3D structures of two molecules (receptor and ligand) that bind to each other by simulating interacting surfaces and free energy minimization at the domain level [34]. Docking problem requires modeling of surfaces using spheres (or grids) and identifying the best match that will fit two surfaces without excessive intersection. Many times biochemical information such as binding sites is provided. There are three major problems in docking: (i) for multi-domain proteins conformation may change during docking, (ii) docking algorithms have high computational overhead that makes large-scale modeling quite slow, and (iii) docking algorithms suffer from over prediction that results in a high number of false positives.

### Pair-wise genome comparison

After the identification of gene-functions, a natural step is to perform pair-wise genome comparisons. Pair-wise genome comparison of a genome against itself provides the details of paralogous genes – duplicated genes that have similar sequence with some variation in function. Pair-wise genome comparisons of a genome against other genomes have been used to identify a wealth of information such as ortholologous genes – functionally equivalent genes diverged in two genomes due to speciation, different types of gene-groups – adjacent genes that are constrained to occur in close proximity due to their involvement in some common higher level function, lateral gene-transfer – gene transfer from a microorganism that is evolutionary distant, gene-fusion/gene-fission, gene-group duplication, gene-duplication, and difference analysis to identify genes specific to a group of genomes such as pathogens, and conserved genes [11,13].

To derive orthologs and sets of gene-groups, genomes are modeled as an ordered set of genes, and a pair of genomes is modeled as a bipartite graph where each node in one set is connected to homologous nodes – similar genes using pair-wise gene-alignment – in the second set. Orthologs are derived as the best matching homologs. To identify homologous gene-group, two neighboring genes in one genome that are homologous to two neighboring genes in the other genome are identified, a window consisting of neighboring genes is created in both the genomes and slided until the next gene in the first genome has no homologous gene in the corresponding neighborhood window in the second genome. After a non-matching gene is identified, the matching genes are collected as one gene-group.

The detailed comparative study [11,12,14] has shown that: (i) a large percentage of these gene-groups are co-transcribed or co-regulated [11,26], (ii) there are multiple types of gene-groups in a genome, (iii) the order of homologous genes in a gene-group is not always the same in two microorganisms, (iv) gene-groups are duplicated a lot, (v) all the genes in ordered gene-group are embedded in the same pathway, and unordered gene-groups occur at the junction points of adjacent pathways [12], (vi) larger genomes share more genes-groups despite not being evolutionary too close, (vii) gene-duplication and gene-insertion/gene-deletion are common means of genome restructuring, and horizontal gene-transfer and gene fusion are not uncommon, and (viii) gene duplication occurs mainly for the genes involved in cell surface interaction, nutrient transport, and sensor proteins. The rationale for duplication is a need to adapt under different external conditions and the use of similar mechanism for multiple sensors and transport proteins. The knowledge of genes specific to pathogens, genes inserted/deleted from pathways that are homologous to genes in the plasmids, and conserved genes are very useful to identify candidates for vaccine development and anti-microbial agents [11,56,73].

An interesting observation of pair-wise genome comparison studies has been that genome restructuring occurs by a combination of insertion/deletion, duplication, and fusion of domains as well as genes. However, the domain level comparative analysis tools are in the stage of infancy due to computational complexity and the limited availability of domain level functional information about various genes from the wet-labs.

### Reconstructing metabolic pathways

Identification of gene functionality has started a new level of bioinformatics research: automated reconstruction and comparison of pathways of newly sequence organisms [12,14,18,38,49,50,58,59,65]. There have been many efforts and approaches related to pathway reconstruction. The three major approaches can be classified as: (i) global network of reactions catalyzed by enzymes, (2) network of gene-groups connected through the reactions catalyzed by enzymes embedded in the gene-groups, (3) global modeling of chemical reactions in the microbial cells.

The first approach [49] uses the knowledge of known biochemical pathways and enzymes [9,33], identifies the enzyme function of new genes in a newly sequenced genome using BLAST based search or using pair-wise genome comparison of evolutionary close genomes [65], and matches the product and substrate of chemical reactions catalyzed by enzymes to build the network of reactions [18]. This approach is quite powerful. However, it has many drawbacks: (i) it can not disambiguate the exact position in pathways for homologous genes, (ii) it does not take into account genes occurring in the same pathway due to gene-grouping and co-transcription, and (iii) it does not take into account the reaction rate.

The knowledge of gene-groups [11,26] has been used to develop an integrated approach for reconstructing metabolic pathways [12,14,65]. In this approach there are four steps: (i) identifying the enzymes and their functions in a newly sequenced genome using ortholog analysis, (ii) identifying the co-transcribed gene-groups – groups of genes sharing a common promoter – by analyzing the promoter region of the genes, (iii) deriving the gene-groups by pair-wise comparison of newly sequenced genome with multiple genomes, and (iv) using biochemical knowledge of existing pathways and enzymes [9,33] to connect network of gene-groups. The intergenic distance – distance between the stop codons of the preceding gene and the start codons of the following gene – in co-transcribed gene-groups (possible operons) is generally less than 75 nucleotides except for the leading gene. By computationally comparing the intergenic distance most of these possible co-transcribed gene-groups are identified. However, the knowledge of co-transcribed gene-groups in itself is insufficient to identify pathways since (i) co-transcribed gene-groups may have missing genes due to conservative estimate of cutoff threshold, (ii) multiple adjacent co-transcribed gene-groups in the same pathway may be separated due to gene insertion/deletion caused by genome restructuring, and (iii) some of the regulating genes that regulate pathways and are in close proximity are not picked up. These three problems are reduced by taking union of genes in the same gene-group derived from multiple pair-wise genome comparisons with the newly sequenced genome. The overall gene-groups are identified by merging the information derived from promoter based analysis and pair-wise genome comparison analysis [14]. Since gene-groups in a pathway are scattered across the genome, the gene-groups are networked to each other by matching the biochemical product and substrates in the reactions catalyzed by the enzymes embedded in the gene-groups using enzyme databases [9,33]. This scheme improves the computational efficiency, reduces the ambiguity of homologous genes, and includes many regulatory genes involved with a pathway. However, this scheme does not model cell level behavior as the notion of reaction rate is missing.

The third approach [50,58] is based on modeling the biochemical reactions globally involving products, byproducts and the effect of cofactors on the reaction rate [59]. The model is based upon representing the network of metabolic reaction as a set of vector of reactions called *extreme pathways *that correspond to study state flux distribution in a metabolic network needed to synthesize target products. In this technique the whole network of pathways is modeled as a matrix where the rows are extreme pathways and columns represent specific reactions. This technique is useful to model the overall metabolic behavior within a microbial cell.

Current metabolic pathway techniques are limited by the available gene-functions from wet-laboratories. Another issue is that the identification of metabolic pathways is not sufficient unless the reaction rates and the effect of stress response over the reaction rates are known. While there have been recent approaches to model the reaction rate of metabolic pathways [59], the complete picture cannot be verified largely due to unavailability of gene-functions from wet-labs.

### Phenotype similarity and automated pathway comparisons

The next level of study that the researchers have taken is to compare the similar pathways to understand the effect of insertion and deletion of genes in various microorganisms and to understand the evolution at pathway level [38]. To compare two pathways, the genes in the pathway are aligned as follows. Two pathways match completely if every protein in the first pathway (or a gene-group within a pathway) has a corresponding homologous gene in another pathway (or the gene-group within the pathway). There is a gap if a homologous gene is deleted (inserted), and there is a mismatch if the corresponding homologous genes have a low similarity score. Based upon this modeling, comparison of *H. pylori *and *yeast *has shown many similar pathways. More importantly, a quantification mechanism has been found to compare two pathways.

### Derivation of regulatory mechanism and pathways

The genomics and proteomics research front has progressively moved from metabolic pathway reconstruction to the identification of signaling pathways and promoter analysis to identify transcription factors for protein-DNA interactions.

There are four major approaches to study protein-DNA interactions: (i) micro-array analysis of gene-expressions under different stress conditions of cells, (ii) statistical analysis of promoter regions of orthologous genes (functionally equivalent genes in different organisms identified as best homologs), (iii) global analysis of frequency patterns of dimers in the intergenic region – promoter region occurring between adjacent protein coding regions – of a genome, and (iv) biochemical modeling at the atomic bond level to understand how a protein will bind to nucleotides. Only the microarray analysis technique is based upon experimental data, and other three approaches are based on mathematical modeling and sequence analysis.

Micro array analysis [69] measures the relative change in the gene-expressions for a stressed (or a stimulated) cell and a change in cellular expression pattern – differentiation, cellular cycle, tissue remodeling, sporulation etc – in response to change in stimuli using a two step process: (i) mapping all the genes in the same genome etched on a thin glass plate and hybridizing the genes of a healthy cell with etched genes to derive the regular gene expression under equilibrium condition, and (ii) hybridizing the affected cells with etched genes to derive the gene expression of affected cells under equilibrium condition. Comparative study of gene expressions under normal condition and under a stimulated (or stressed) condition provides the information about the affected genes. Under the assumption that auto regulation in a gene-group and any cyclic self-regulation is absent, the interaction between protein and transcription factors is responsible for the observed increase or decrease of gene-expressions. This gene-expression data is analyzed using (i) cluster analysis [69] to identify meaningful patterns of gene-expressions, or (ii) data mining techniques – a statistical technique that associates and correlates expressed genes and different stress conditions.

The second approach of statistical promoter analysis [30,43,44] first identifies the orthologous genes from evolutionary close microorganisms [11] with active pathways using pair-wise genome comparisons databases (see ) or using the knowledge of cluster of orthologs (COGS) – a group of genes in a super family archived at NCBI at NIH that has been derived by multiple genome comparisons. In the next step, the upstream region between two genes of the orthologs are identified and compared to identify statistically conserved patterns. Under the assumption that functionally equivalent genes in the very similar pathways of evolutionary close organisms will have similar regulation mechanism, the transcription factors – regions of promoters involved in enhancing or repressing the gene-expression of the associated gene – for protein-DNA interaction in the promoters of orthologous genes would also be very similar. This analysis has led to discovery of many transcription factors.

The third approach [47] has been to extract and statistically analyze the dimers in the intergenic region in a whole genome and plot the frequency of occurrence. The non-random dimers that occur more frequently are possibly involved in protein-DNA interactions.

The biochemical approach [42] studies the protein-DNA interactions at the atomic bond level by considering hydrogen bonds in amino-acid base interactions, Van der Wall forces at contacts and water mediated bonds at different levels of proximity of two molecules. Based upon the analysis of the bonds and the actual statistical results, it has been concluded that amino-acid base interaction plays a major role in binding, Van der Wall forces provide stabilization, and protein-DNA interactions are complex and biased: different amino-acids have preferences for certain types of bases. For example, *arginine*, *lysine*, *histidine *and *serine *have preference for *guanine*.

Currently no researcher has attempted a hybrid approach integrating biochemical approach with other four approaches. An integrated approach will give a better overall picture. Another complex problem is that a co-regulated gene may have more than one transcription factor; some of these transcription factors may be individually weak and may be correlated with other transcription factors. An approach to identify the weak transcription factor is a two step process: (i) first identify the strong related transcription factor using one of the previous approaches followed by (ii) a pattern search in the neighborhood of the strong pattern [27].

Figuring out the connectivity in protein-protein interactions to derive signaling pathway has been a long drawn challenge. Recently, in last two years, two approaches have emerged: (1) integration of microarray analysis and entropy based modeling to derive gene clustering of the genes involved in the same regulatory pathway [2,7], and (2) technique based upon random algorithms maximizing transition probability. The first approach computes the mutual information of all the gene-pairs, and clusters the protein groups having more mutual information above a threshold [20]. The mutual information is entropy based approach, and is derived by the cumulative sum of the frequency patterns of occurrence of gene-pairs. To derive entropy, gene-expressions are divided into discrete histograms, and the mutual information between every gene-pair is computed [20]. Higher mutual information means direct correlation of the genes. It has been statistically found that genes that belong to the same pathway tend to group together. Using this cluster analysis, many signaling pathways have been identified in yeast-based system [15]. The analysis is a general-purpose technique, and can be used both in prokaryotic as well as eukaryotic systems.

Even figuring out the connectivity will not be able to answer the transient temporal behavior of many genes involved in the regulation mechanism and auto-regulation mechanism of operons – co-transcribed gene-group within a pathway involved in a common functionality. The modeling of transient behavior of genes cannot be captured by hybridization based microarray analysis since the data corresponds to equilibrium state of reactions. To understand the malfunctioning cells and cells of pathogenic bacterial strains, the overall organization and behavior including transient behavior and stress responses have to be studied.

### Microbial evolution revisited

Bioinformatics researchers have compared extensively multiple genomes to correlate and classify the genomes into various families and to study evolution. It has been established by many researchers that overall evolution is a combination of point based mutation giving rise to speciation and restructuring of genomes based upon gene duplications, gene insertion, gene deletion, gene-fusion/fission, horizontal gene transfer, and domain level restructuring [11,17,45].

The evolutionary study efforts can be classified into three approaches: (1) point based mutation approaches used to build traditional evolutionary tree using multiple sequence alignment of 16SrRNA [72], (2) study of genome restructuring based upon inversion and transposition at the gene level [17,45], and (3) the study based upon whole genome comparisons using gene identity of orthologous genes across multiple microbial genomes [13].

The 16SrRNA approach is rooted in the concept of point mutation of conserved genes due to their slow mutation rate, uses 16SrRNA database and multiple sequence alignment [22], and uses neighbor join algorithm [36] to build an evolutionary tree. Before microbial genomes were sequenced, this technique was considered quantitatively sound, and using 16SrRNA database three distinct domains – bacteria, archaea, and eukaryotes – were identified. Archea domain is hyperthermophilic, and its 16SrRNA is somewhat different from 16SrRNA of bacteria.

Since 1998, after the availability of multiple microbial genomes, the researchers have tried to build the evolutionary tree by comparing other highly conserved genes. The results have shown that the evolutionary tree varies a lot depending upon the choice of the conserved genes, and shows no clear distinction between archaea and bacteria. This observation combined with the knowledge of genome restructuring caused by domain level and gene level restructuring such as horizontal gene transfer has shaken up the traditional evolutionary trees based upon point mutations in 16SrRNA [54].

The second approach uses the genome rearrangement caused by gene shuffling as a measure for the genomic distance between the two organisms [17,45]. Gene shuffling is caused by inversion and transposition. This scheme is based upon the distance measure as the breakaway from the standard gene-order in two genomes. Under this scheme the breakaway distance for each orthologous gene is added to give a cumulative score for the genome. This score is used as a distance between two genomes. Building large scale evolutionary tree using this approach was cost prohibitive due to pair-wise comparison until recently when a new development in parallel algorithms made such an evolutionary tree possible [45]. Is this scheme horizontal transfer of genes do not play a role: insertion and deletion are not counted in the assumption, and duplications are mapped to a single gene. It has been shown that duplication, insertion, deletion of gene-domains and genes are a major component of evolution [11]. Specially duplicated genes are involved in multiple sensor and transportation pathways such as ABC transporters, and cannot be ignored.

The third approach [13] is based on comparing overall gene-content of functionally equivalent genes to identify the cumulative similarity of two genomes. The data is normalized to take care of different size of genomes. The major assumption in this scheme is that conserved genes are very few and do not give a consensus, and slow mutation rate only contributes to good multiple sequence alignment. Whole genome comparisons can balance out the error introduced by comparing a single conserved gene. The results show that the overall amino-acid composition in the microorganisms does not differ significantly between archaea and bacteria to give a separate domain status to archaea [13]. In addition, the composition of other hyperthermophilic bacteria cannot be distinguished from archaea.

Currently no proteomic level approach has been suggested to classify the genomes. In future, one such approach could be based upon comparative analysis and alignment of pathways of multiple genomes [38]. Under this scheme, after the pathways are aligned, a combination of the cumulative number insertion and deletion of genes in the pathways, gene duplication in the same pathway, and gene shuffling could be used to describe the distance between two genomes since all three factors are directly involved in the pathway variations. However, exact mechanism of combining these three components of pathway evolution has to be studied.

## Conclusion

Despite being a young field, bioinformatics has helped both fundamental microbiology and biotechnology through the development of algorithms, tools, and discoveries refining the abstract model of microbial cell functioning. The major impact of the bioinformatics has been in automating the microbial genome sequencing, the development of integrated databases over the Internet, and analysis of genomes to understand gene and genome function. BLAST based database search and Smith-Waterman based gene-pair alignment algorithm and their variations are being used extensively in comparing genes and genomes, and have become the first steps to derive the gene-function and the functionality of genomes. Significant success has been achieved in comparative genome analysis to: (i) identify conserved function within a genome family, (ii) identify specific genes in a group of genomes, and (iii) model 3D structures of proteins and docking of biochemical compounds and receptors. These successes have direct impact in the development of anti microbial agents, vaccines, and rational drug design. By integrating the knowledge of orthologs and gene-functions, gene-grouping based upon the integration of pair-wise genome comparison, and co-transcribed gene-groups, and graph based matching of substrates and products catalyzed by enzymes metabolic pathways reconstruction has been nearly automated.

The current front has moved to the identification of regulatory pathways, identification of protein-protein interactions, protein-DNA interactions, protein-RNA interactions, and simulations of metabolic reactions to study the effect of reaction rates, and the analysis of experimental data available from micro-array data to study the correlation between the gene-expressions and stress conditions.

Most of the bioinformatics techniques are critically dependent upon the knowledge derived from wet laboratories and the available computational algorithms and tools. Unfortunately, both the resources have limited capability of handling a vast amount of data to interpret genomics and proteomics with so many unknowns. Since there is a limited set of gene-functions available from the wet lab data, there are many holes in the complete picture of gene functions in many newly sequenced genomes. A lack of integration of bioinformatics research with biochemical knowledge also contributes to the holes in the complete picture.

The mathematical modeling approaches are suitable for new discoveries to derive candidate genes for vaccine and rational drug design, metabolic pathways, metabolic pathway variations, and transcription factors for regulatory pathways. However modeling results contain many false positives and false negatives. These results need to be verified and cured by wet-lab experiments. However, complete verification is becoming humanly impossible due to the unavailability of experts, resources, and problems in co-ordination and ever changing bioinformatics databases caused by new analysis and discoveries [51].

With the availability of better cell visualization techniques and the abstract genomics models based upon current bioinformatics analysis and their integration with existing biochemical knowledge, the microbial wet lab experiments will become more focused in their goal. The progress in bioinformatics and wet-lab techniques has to remain interdependent and focused complementing each other for their own progress and for the progress of biotechnology in future. In future more and more focus would be to apply the techniques in an integrated way to manipulate the microbial cells at systemic level.

## Authors' contributions

AB is the sole contributor of this original review article. The review is based upon the published current research in the area of microbial bioinformatics.
